# Trends and Determinants of Oral Anti-Diabetic Initiation in Youth with Suspected Type 2 Diabetes

**DOI:** 10.1371/journal.pone.0140611

**Published:** 2015-10-28

**Authors:** Mona Cai, Michael D. Kappelman, Cynthia J. Girman, Nina Jain, Til Stürmer, Maurice Alan Brookhart

**Affiliations:** 1 Department of Epidemiology, UNC Gillings School of Global Public Health, Chapel Hill, NC, United States of America; 2 Department of Pediatrics, Division of Pediatric Gastroenterology, UNC School of Medicine, Chapel Hill, NC, United States of America; 3 CERobs Consulting, LLC, Chapel Hill, NC, United States of America; 4 Department of Pediatrics, Division of Pediatric Endocrinology, UNC School of Medicine, Chapel Hill, NC, United States of America; McMaster University, CANADA

## Abstract

**Objective:**

To evaluate trends and identify predictors of treatment initiation of oral anti-diabetic drugs (OAD) in youth.

**Patients and Methods:**

We identified a select population of children, ages 8–18 years, with at least 13 months of continuous health plan coverage within the years 2001–2012 in a large US commercial insurance claims database. New use of an OAD was defined as the first claim for an outpatient dispensing following a 12-month wash out period. Treatment incidence was estimated monthly over the study period, and stratified by age, gender, geographic region, and provider specialty.

**Results:**

The median size of the source population during the study period was 2.2 million children. A total of 13,824 initiators (mean monthly incidence of 4.6 (95% CI = 3.6, 5.5) per 100,000 youths) were identified. Initiators were more likely to be females, age 15–18, from the southern region, and have visited a family practitioner (versus a general pediatrician) prior to initiation. Time trends demonstrate a 43% increase in initiation from 2002–2012, with a gradual decrease starting from early 2008.

**Conclusion:**

Incidence of filled OAD medications in youth increased over time, especially for patients treated by family practitioners. Additional research is needed into factors influencing prescribing by family practitioners and pediatricians.

## Introduction

The rise in obesity among children and adolescents is well documented in the literature and has become an established public health concern[1,2]. The high burden of obesity has paralleled the growing prevalence of metabolic syndrome (MS)-related comorbidities such as hypertension, type 2 diabetes mellitus (T2DM), and hyperlipidemia, which were widely regarded as adult-onset conditions until recent decades[3]. T2DM is the most common form of diabetes in adults, comprising over 90% of all diabetes and affecting roughly 8.3% or approximately 21 million of all adults in 2010[4,5]. As of year 2006 the prevalence amongst youth was much lower than adults at an estimated 0.2%[6]; however, the prevalence of prediabetes was 16.1%[7], which if left improperly managed in adolescents can progress to T2DM in as little as 3 years[8]. Incidence data from 2002–2003 reported rates ranging as high as 49.4 per 100,000 person-years amongst Native Americans to 5.6 per 100,000 person-years amongst non-Hispanic whites, with an approximately 60% higher rate in females than males across ethnic groups[9]. Some projections suggest that the number of T2DM cases will quadruple by 2050 if T2DM incidence increases by 2.3% yearly[10]. Earlier age onset of T2DM increases the likelihood of lifetime incidence of numerous chronic comorbidities and consequentially, increasing morbidity and mortality[11]. There will be countless economical and public health challenges in the future as these patients transition into early adulthood.

Treatment guidelines pertaining to youth, first released by the American Diabetes Association (ADA) in 2000, include both screening and therapeutic recommendations[12–14]. Metformin is currently the only oral anti-diabetic (OAD) agent approved by the FDA for treatment of T2DM in youth ≥10 years of age; however, off-label use of OADs in youth has been reported to be common[12]. Over the years, advancements in guidelines and intensifying public awareness have led to increased T2DM diagnosis and implementation of more aggressive treatment regiments. This along with the overall growing number of prediabetes and T2DM cases has encouraged the introduction of T2DM therapeutics into this population, though practice patterns may vary both between and within provider specialties.

The chronological trends and predictors in treatment initiation of OADs are not well documented. This study utilized longitudinal prescription claims data sampled from a population of US commercially insured children and adolescents to assess the temporal trends in filled OAD prescriptions and examine the association between patient and prescriber characteristics and treatment initiation. We hypothesized that the incidence of OAD initiation had increased over the course of the study period, with variations in prescription rates by provider specialty.

## Materials and Methods

### Data Source

Using the Marketscan™ Research Database, we studied a population of youth enrolled in an employer-provided private insurance plan between the years 2001 to 2012. Marketscan is comprised of a large and diverse sample of the U.S. commercially insured population and contains comprehensive individual-level records on patient demographics, enrollment information, inpatient, outpatient, and prescription drug claims[15]. In 2012, the database included approximately 5.5 million youth aged 6–17, equivalent to 10% of the overall population and 20% of the commercially insured population in the US for that age group[16,17].

### Patient Population

Patients aged 8–18 years newly initiated on a therapy from any class of OADs (metformin, sulfonylureas, thiazolidinediones, alpha-glucosidase, meglitinide analogs, glucagon-like peptide-1 agonist, and dipeptidyl peptidase-4 inhibitors) during the index period of January 2002-Decemeber 2012 were identified. Patients on insulin only without evidence of OADs were excluded. “New users” were defined based on the following criterion: (1) patients had ≥12-months of continuous enrollment prior to their oral drug fill date and (2) patients without an OAD medication in the 12-months prior to their drug fill date, hereon referred to as their “index drug” date. Youth likely to have any of the following diagnoses, as indicated by ICD-9 codes associated with inpatient or outpatient claims, were excluded from the study population: type 1 diabetes (ICD-9: 250.x1, 250.x3), gestational diabetes (ICD-9: 648.8), and females with polycystic ovarian syndrome (PCOS) (ICD-9: 256.4. Furthermore, females with diagnosis codes for symptoms of PCOS including hirsutism (ICD-9: 704.1) and ovarian cysts (ICD-9 620.0 and 620.2) were also excluded.

### Predictors of Treatment Initiation

Potential predictors of treatment initiation were determined at baseline and included age (age groups: 8–10, 11–14, and 15–18), gender, the U.S geographic region (Northeast, North Central, South, and West) that the patient resided in, and physician specialty (family practice, pediatrician, both, and neither). The defined age categories considered that young children are off-label users, and early teen versus late-teen initiation rates may vary as T2DM disproportionately affects late-teens, e.g. 15–19 year olds [18]. Physician specialty was categorized based on those who had at least one visit to a pediatrician, family practitioner (FP), both a pediatrician and FP, and neither a pediatrician nor FP in the three-months prior to their index date. Analyses utilizing a six-month physician visit window were also performed to evaluate the impact of the pre-defined window on study results. All other predictors were pre-categorized in the Marketscan database.

### Statistical Analysis

Treatment incidence among children and adolescents initiating OAD therapy was estimated monthly from January 1, 2002 through December 31, 2012. Rates for each month of the study period were calculated by dividing the number of eligible patients with an index date falling in that month by the total number of youth who would have been in the numerator had they filled their index prescription in that month, i.e. age-eligible persons continuously enrolled in the 12-months prior who did not satisfy any exclusion criteria. Initiators were omitted from the denominator in the subsequent months after their index date.

Descriptive analyses included mean monthly frequencies for baseline characteristics, reported separately for the general denominator and the new user populations, and mean monthly incidences. Mean monthly incidences and 95% CIs were calculated per 100,000 youth per month and were reported for the entire population as well as by explanatory variables. In order to evaluate the strength of *a priori* identified predictors, mean monthly relative risks (RR) and 95% CIs were calculated and assessed over calendar time. Trends in prescription rates, smoothed using local polynomial regression[19], were graphed along with 95% confidence intervals (CIs) monthly for the overall population as well as for each predictor subgroup. The physician specialty graph included plots for the subgroups of patients who only visited a pediatrician or only visited a FP in the 3-months prior to their index date. All analyses reported monthly means for the calendar periods of 2002–2012, 2002–2003, 2004–2005, 2006–2007, 2008–2009, and 2010–2012.

## Results

The average monthly population size increased 6-fold over the course of the study period (Table 1) as the Marketscan Research Database increased in size over that time from 700,000 to over 4 million individuals, with a median population size of 2.2 million. Frequency distributions in age, sex, and geographic region remained consistent in the population sample throughout the study period, while minor variations in physician specialty encounters were recorded, signifying a small increase in primary care utilization by this population (Table 1).

**Table 1:** Beneficiary Baseline Characteristics By Study Period.

Characteristic	Study Period				
	2002–2003	2004–2005	2006–2007	2008–2009	2010–2012
Mean population size per month N (SD)	783 890(225 623)	1 704 762(286 599)	1 975 504(200 922)	2 661 589(530 239)	3 393 037(464 836)
Age (%)					
8–10	23.5	24.1	24.2	24.8	24.9
11–14	36.5	36.3	35.8	35.7	36.1
15–18	40.0	39.6	40.0	39.5	38.9
Gender (%)					
Female	48.7	48.7	48.8	48.8	48.8
Region (%)					
NE	13.2	9.3	11.1	10.1	13.9
NC	28.6	24.0	27.3	26.9	26.4
South	38.3	37.9	41.9	43.6	39.8
West	20.0	28.7	19.7	19.4	19.9
Physician Specialty^a^ (%)					
Family practice	8.4	9.5	10.2	10.2	8.7
Pediatrician	13.3	14.5	16.8	18.1	18.6
Both	0.5	0.6	0.7	0.8	0.8
Neither	77.9	75.4	72.3	70.9	71.9

SD, Standard deviation; NE, Northeast; NC, North Central

^a^ Mean monthly percentage of population with medical encounters in the 3-months prior

A total of 13,824 new users of any OAD prescriptions were identified between 2002 and 2012 (Table 2). Baseline characteristics of OAD initiators fluctuated slightly over the years as the percentage of females, 8–10 year olds, and patients from southern region increased by 4.7%, 13.7%, and 13.8%, respectively, from 2002–2003 to 2010–2012. Notable decreases were seen in the 15–18 year olds (6.1%) and patients from the Northeastern (15.1%) and Western (19.6%) regions. Metformin was the most commonly initiated OAD drug class overall (88.6%) and showed a 21.2% increase in usage from 2002–2003 to 2010–2012. Usage of all other drug classes went down from 21.1% to 4.4% by the end of the study period. The proportion of new users who visited a pediatrician or FP in the 3-months prior to index date from 2002–2003 to 2010–2012 increased from 19.2 to 25.4% or 20.5 to 24.1%, respectively.

**Table 2:** Baseline Characteristics of New Users of Oral Anti-Diabetic Agents by Study Period.

Characteristic	Study Period				
	2002-2003(n = 616)	2004-2005(n = 1784)	2006-2007(n = 2429)	2008-2009(n = 3246)	2010-2012(n = 5749)
Age (%)					
8–10	7.3	8.5	10.2	8.9	8.3
11–14	30.7	32.2	32.8	33.6	33.5
15–18	62.0	59.4	57.0	57.6	58.2
Gender (%)					
Female	68.5	67.5	70.0	70.6	71.7
Region (%)					
NE	13.9	7.3	7.3	6.4	11.8
NC	26.4	25.4	27.7	29.3	26.9
South	39.8	45.2	51.7	49.8	45.3
West	19.9	22.0	13.3	14.4	16.0
Index Drug Type (%)					
Metformin	78.9	84.9	89.4	94.5	95.6
Sulfonylurea	6.0	5.8	3.5	1.9	1.5
TZD	8.9	4.5	3.5	1.2	0.6
Other Classes^a^	1.8	1.6	2.3	1.2	1.8
Metformin + Sulfonylurea	3.6	1.4	0.7	0.6	0.3
Metformin+ TZD	0.8	1.8	0.5	0.5	0.2
Physician Specialty^b^					
Family Practice	20.5	24.2	25.4	26.1	24.1
Pediatrician	19.2	21.8	24.7	24.3	25.4
Both	4.3	3.6	2.5	3.2	2.8
Neither	54.7	51.3	47.6	46.7	47.8

NE, Northeast; NC, North Central; TZD, Thiazolidinediones

^a^ Includes alpha-glucosidase, meglitinide analogs, glucagon-like peptide-1 agonist, and dipeptidyl peptidase-4 inhibitors

^b^ Based on medical encounters in the 3-months prior to index drug date

The overall monthly incidence in the population was 4.6 (95% CI = 3.6, 5.5) per 100,000 youths (Table 3). Patients who initiated treatment were more likely to be females (RR = 2.57; 95% CI = 1.59, 4.39), age 15 to 18 (8–10 years, RR = 4.74; 95% CI = 2.04, 13.60; 11–14 years, RR = 1.69; 95% CI = 1.03, 2.90), and residents of the southern region (Northeast, RR = 1.91; 95% CI = 0.77, 6.02); North Central, RR = 1.22; 95% CI = 0.70, 2.31; West, RR = 1.72; 95% CI = 0.88, 3.85). They were also twice as likely to have visited a family practitioner, compared to a general pediatrician, in the 3-months prior to OAD initiation (RR = 2.00; 95% CI = 1.02, 5.02).

**Table 3:** Monthly Mean Incidence (95% CI) Per 100 000 Commercially Insured Children: Overall and by Predictor Subgroups

Characteristic	Study Period					
	2002–2012	2002–2003	2004–2005	2006–2007	2008–2009	2010–2012
Overall	4.6 (3.6, 5.5)	3.3 (2.0, 4.6)	4.4 (3.4, 5.4)	5.1 (4.1, 6.1)	5.1 (4.2, 6.0)	4.8 (4.0, 5.5)
Age						
8–10	1.4 (0.4, 2.3)	1.1 (-0.4, 2.5)	1.3 (0.3, 2.2)	1.8 (0.7, 2.8)	1.4 (0.6, 2.1)	1.3 (0.6, 2.0)
11–14	3.4 (2.1, 4.6)	2.5 (0.7, 4.3)	3.2 (1.9, 4.5)	3.8 (2.5, 5.0)	3.6 (2.5, 4.6)	3.6 (2.6, 4.5)
15–18	5.5 (3.9, 7.0)	4.5 (2.3, 6.8)	5.5 (3.8, 7.1)	5.9 (4.4, 7.4)	5.6 (4.3, 6.9)	5.7 (4.5, 6.9)
Gender						
Female	6.5 (4.9, 8.2)	4.7 (2.4, 6.9)	6.1 (4.4, 7.8)	7.4 (5.6, 9.1)	7.4 (5.9, 8.9)	7.0 (5.7, 8.3)
Male	2.7 (1.6, 3.7)	2.0 (0.6, 3.5)	2.7 (1.6, 3.8)	3.0 (1.9, 4.1)	2.9 (2.0, 3.8)	2.7 (1.9, 3.4)
Region						
NE	3.4 (1.0, 5.8)	2.6 (-0.05, 5.8)	3.5 (0.5, 6.4)	3.4 (1.0, 5.8)	3.3 (1.1, 5.4)	4.0 (2.2, 5.8)
NC	4.7 (2.8, 6.6)	3.3 (0.9, 5.7)	4.6 (2.5, 6.7)	5.4 (3.3, 7.2)	5.6 (3.8, 7.4)	4.9 (3.4, 6.3)
South	5.5 (3.8, 7.1)	4.1 (1.8, 6.5)	5.3 (3.5, 7.1)	6.4 (4.7, 8.2)	5.9 (4.5, 7.3)	5.5 (4.2, 6.8)
West	3.6 (1.7, 5.5)	3.1 (0.1, 6.2)	3.3 (1.7, 5.0)	3.5 (1.6, 5.3)	3.8 (2.1, 5.5)	3.9 (2.4, 5.4)
Physician Specialty^a^ ^,^ ^b^						
Family Practice	11.9 (6.8, 17.0)	8.2 (1.2, 15.1)	11.4 (6.1, 16.6)	12.8 (7.8, 17.7)	13.3 (8.9, 17.7)	13.2 (9.1, 17.4)
Pediatrician	6.5 (3.6, 9.3)	4.7 (0.5, 8.9)	6.6 (3.3, 9.8)	7.6 (4.6, 10.5)	6.8 (4.5, 9.2)	6.5 (4.5, 8.5)
Both	2.0 (-0.3, 4.2)	3.0 (-2.1, 8.1)	2.4 (-0.4, 5.3)	1.8 (-0.4, 4.0)	2.0 (0.1, 3.8)	1.6 (0.2, 3.0)

CI, Confidence Interval; NE, Northeastern; NC, North Central

^a^ Based on medical encounters in the 3-months prior to index drug date

^b^ Patients who visited “neither” specialties were omitted from table

### Trends in Incidence of Use

**Fig 1:**
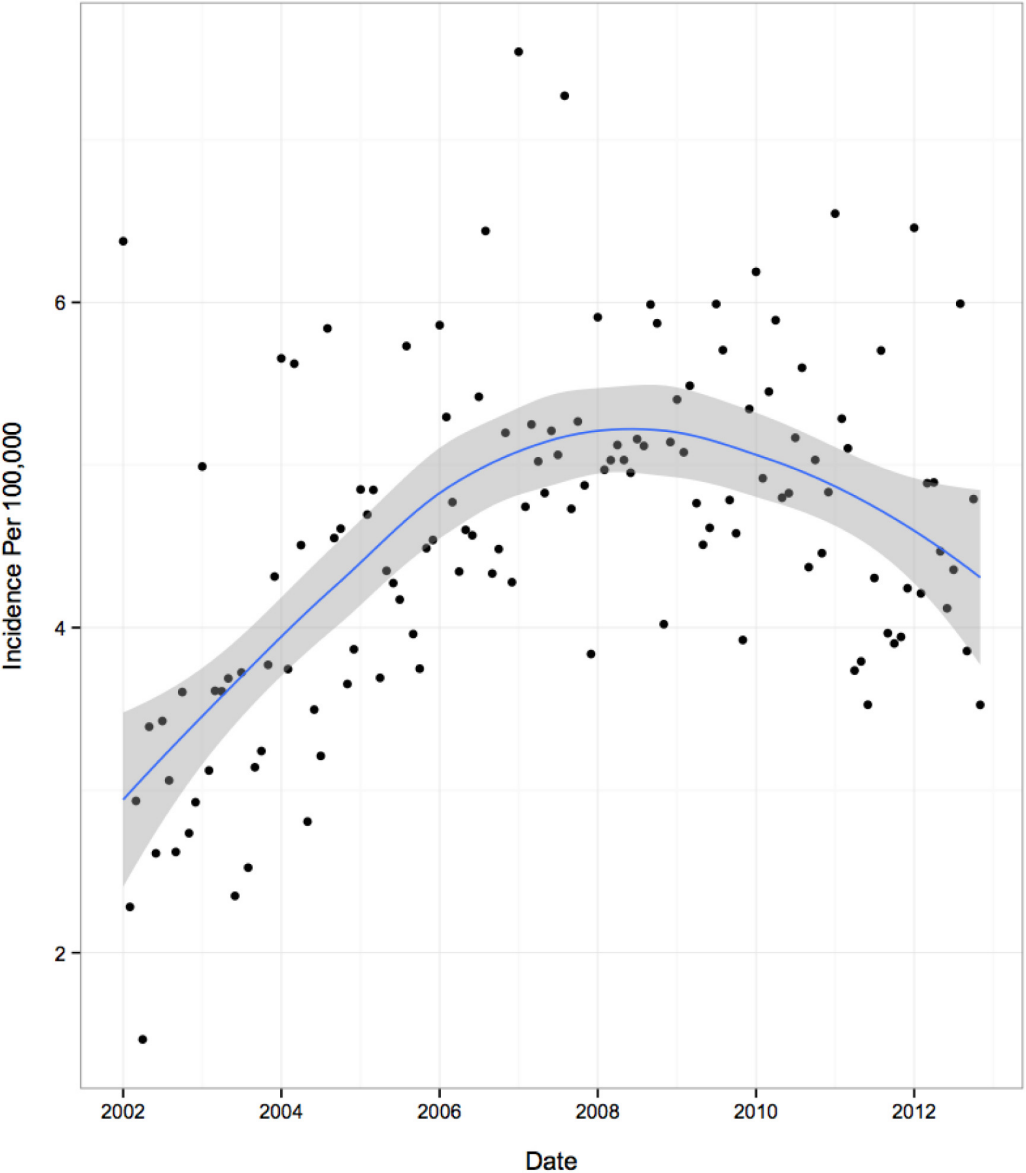
Overall Population Trends: Incidence Trends per 100 000 Youth.

**Fig 2:**
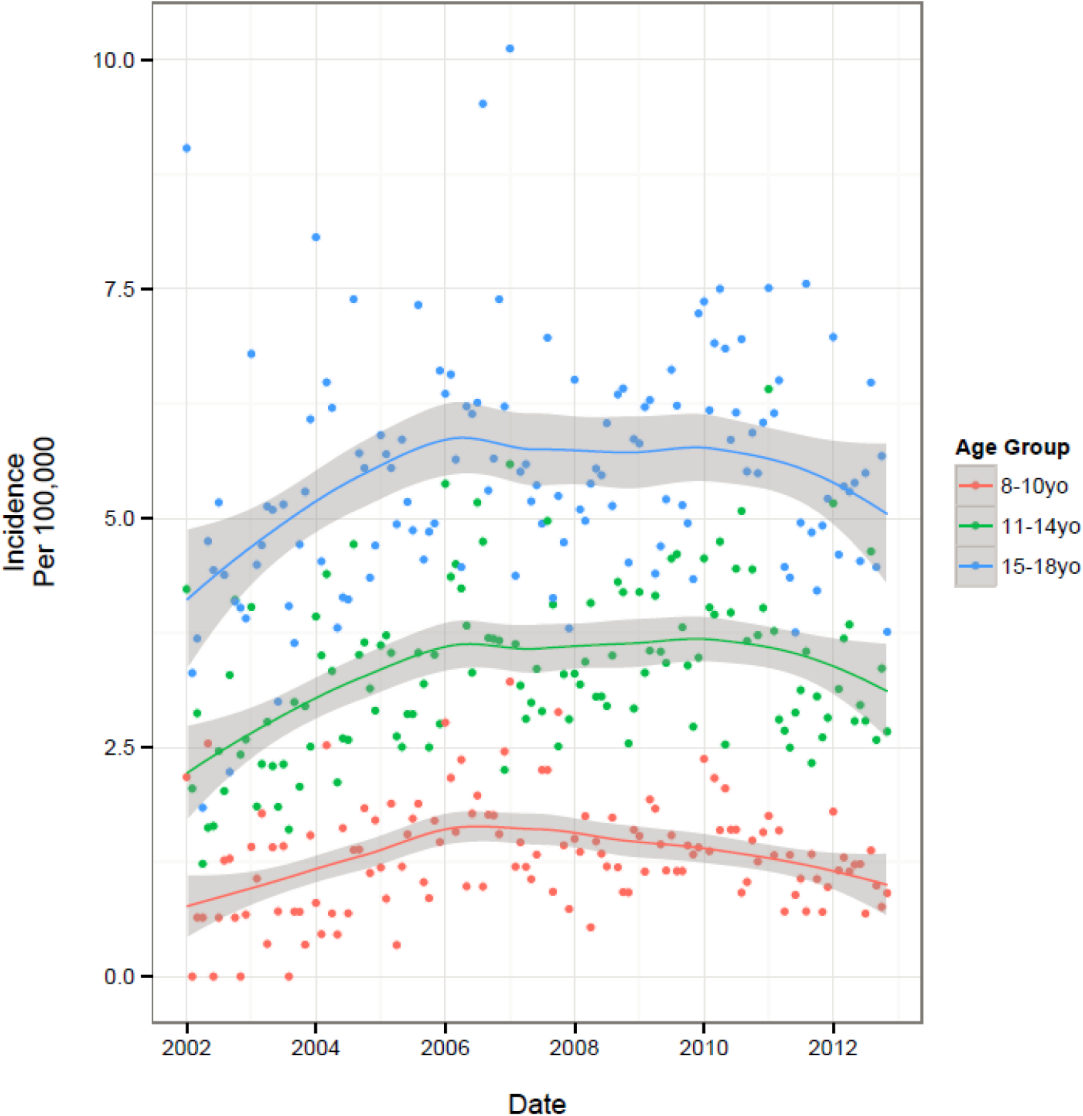
Population Trends by Predictor of Initiation- Age Group Specific Trends: Incidence per 100 000 Youth.

**Fig 3:**
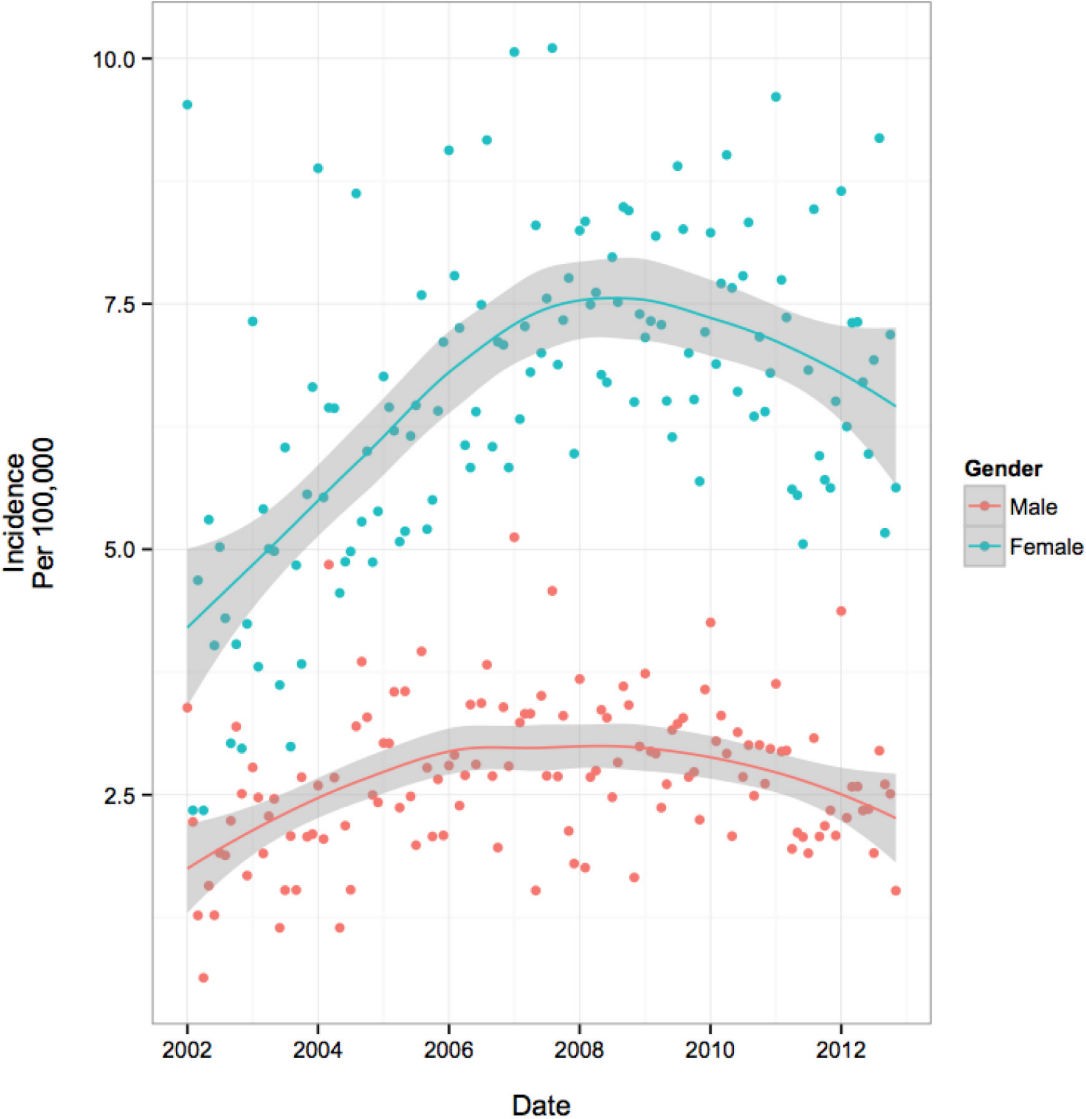
Population Trends by Predictor of Initiation- Gender Specific Trends: Incidence per 100 000 Youth.

**Fig 4:**
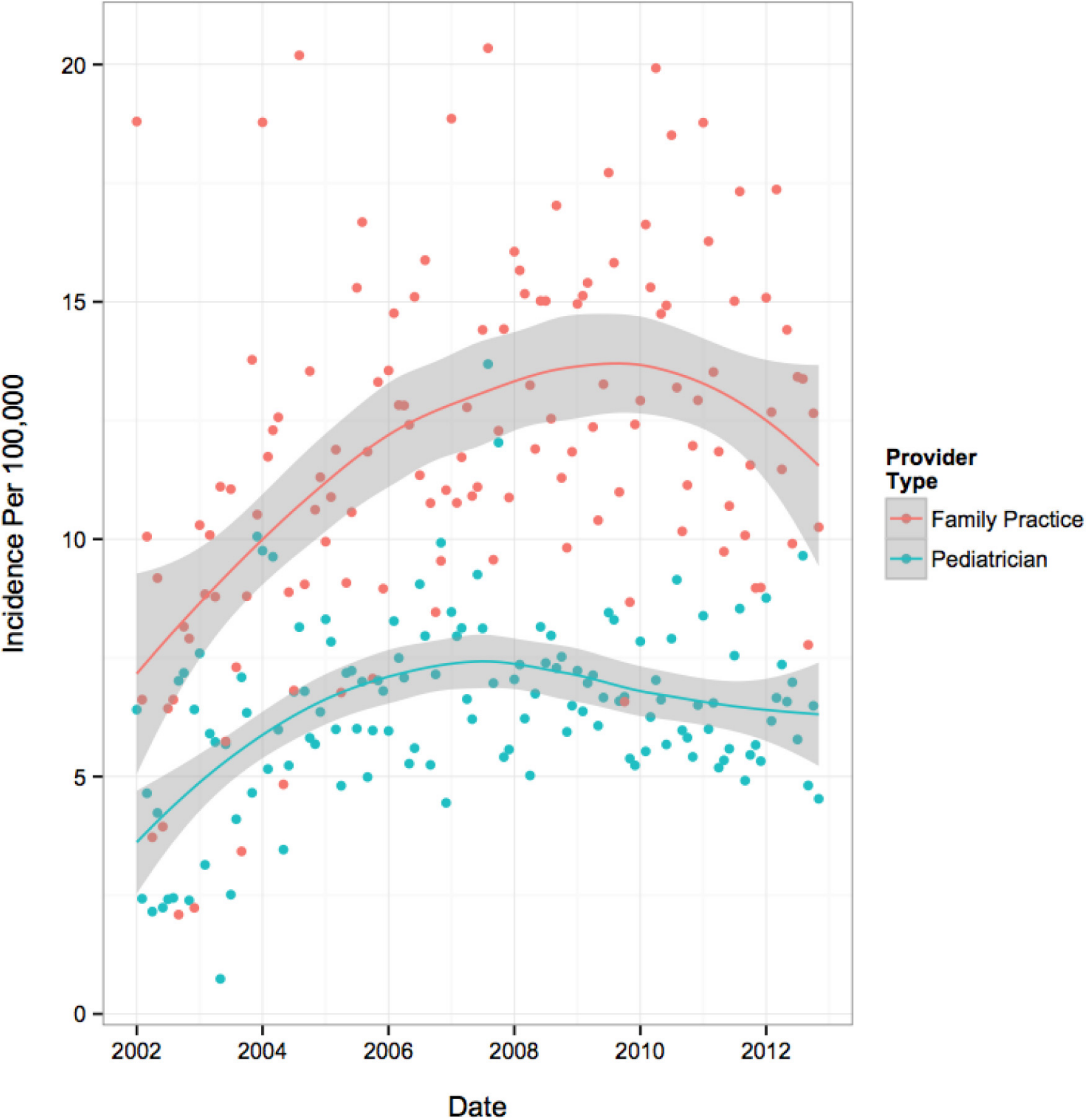
Population Trends by Predictor of Initiation- Physician Specialty Trends: Incidence per 100 000 Youth.

The temporal trends in overall and subgroup specific monthly incidence rates are presented in Table 3 and visually depicted in Figs 1–4. Fig 1 illustrates an increase in overall incidence between years 2002 (3.0 per 100,000 youth) to early 2008 (5.3 per 100,000), before gradually declining during the remainder of the study period (4.3 per 100,000 in 2012), suggesting a corresponding 43% increase in new users over the course of 11 years. This pattern was also reflected consistently in all age group specific trends (Fig 2) and to a lesser extent, the regional trends. Gender-specific initiation rates over time illustrated differences in temporal trends between male and female patients (Fig 3). Female incidence increased by 45% between 2002 to early 2008 before experiencing a 12% decrease for the remainder of the study period. Overall, the female population underwent a 62% increase in usage over 11-years. The male population experienced their peak in incidence 2-years earlier than females in 2006 where rates increased by 67% from 2002. Their overall increase during the study period was 28%, demonstrating a lesser increase compared to their female counterparts. At all time points, individuals with visits to a FP physician were approximately twice as likely to initiate OAD as compared to individuals with visits to a general pediatrician (Fig 4). Individual trends show an 89% increase in FP prescriptions from 2002 to mid-2009 and then dropping by 11% during the remainder of the study. Pediatrician trends suggest a 95% increase in prescriptions from 2002 to early 2007, before experiencing a 14% drop over the course of the remaining 5-years of the study period. Overall, FPs and pediatricians experienced similar percent increases in prescriptions over the duration of the study, 67% and 68%, respectively.

## Discussion

The incidence of filled OAD prescriptions among children and adolescents increased substantially from 2002 to 2012, especially among females and individuals treated by FPs. Temporal trends demonstrate a decline in new prescriptions starting in 2008, counterbalancing the sharp increase in new prescriptions from 2002 to 2008. We estimated similar trends of increasing use followed by gradual declines in treatment initiation for all sub-group specific analyses. The off-label usage of all other OAD drug classes decreased in this population over time as nearly all patients were on metformin by 2008. Conversely, off-label prescriptions for children aged 8–10 increased during the study period.

To our knowledge very little has been published on the incidence of pharmacy dispensed OAD prescriptions in children and adolescents, which makes comparisons with other studies difficult. Two studies were identified that evaluated prevalence trends in OAD prescriptions amongst youth in the US over a period spanning from 2002 to 2007. Conclusions drawn from both studies were consistent and illustrated an approximate doubling in prevalence with the highest prevalence amongst females and adolescents[20]^,^ [21]. The results from our study are consistent with this earlier work, as we reported increasing incidence of use from 2002–2008, with the highest incidence observed in females and patients 15–18 year olds. However, we were unable to evaluate our observed decrease in incidence between 2008 and 2012 with the prevalence trends during that same time period because both aforementioned studies were completed by 2007. Our study significantly extends this prior work by providing estimates for the number of new users of OAD therapies, which allowed us to examine factors influencing treatment initiation.

The trend in T2DM disease incidence and prevalence has been well documented by the SEARCH for Diabetes in Youth Research group[18,22,23]. Based on their reports, the prevalence and incidence of T2DM has increased by 30.5% and 37.5%, respectively, between the years 2001 and 2009 with the most substantial subgroup increases seen in females, late-teens between 15–19 years of age, and racial minorities [18]. The estimated increase in T2DM disease incidence is aligned with our findings on OAD incidence; however, we are unable to correlate our findings with theirs after the year 2009 where we observed a decrease in new OAD fills.

Obesity is the primary risk factor for T2DM, with over 50% of all obese adolescents having clinical markers for the condition[24]. Therefore, the decreasing trend in new prescriptions beginning in 2008 reported by this study may be partially attributed to the fact that the prevalence of obesity has not increased in this population since 2007[25]. However, gender-specific trends are not consistent with obesity trends. Our study found that initiators were more likely to be females, whereas gender-specific obesity rates consistently show higher rates among males[25], making it unlikely that obesity is the only factor explaining the observed trends. It is well-documented in the literature that T2DM rates are consistently higher in females compared with males during adolescence[22,26]. However, one factor that may erroneously inflate the rate of female initiators is PCOS, which is a known risk factor for T2DM and is often treated with metformin. We therefore excluded all patients with a diagnosis for PCOS and PCOS-related symptoms in order to achieve a more homogenous population. Furthermore, we compared the trends in PCOS and symptom-related diagnoses with female initiators over the same calendar period, and established that PCOS was unlikely to impact the observed female trend. We recognize that by excluding PCOS, our study may have somewhat underestimated the true rate of OAD initiation.

Our observation that initiators were twice as likely to have visited a FP compared with a general pediatrician is of particular interest. Obesity and obesity-related comorbidities are difficult to manage, contributing to the low self-perceived competence level to treat such disease states by physicians[27,28]. Differences in attitudes and management of T2DM have been seen to vary by physician characteristics[29]. Results from survey data showed that younger providers and female physicians were more inclined to be aggressive with screening and monitoring practices[29]. Less is known about how provider specialty impacts treatment decisions regarding OADs. However, as FPs frequently manage adult patients with T2DM and pre-diabetes[30,31], they may be more comfortable with prescribing OADs than general pediatricians. Therefore, it is not surprising that our study reported differences in prescribing rates by provider type. Nevertheless, this high degree of variation by provider may indicate overuse and/or underuse, and suggests opportunities for improvement in education, training, and care.

Our study has several limitations. First, our results are not representative of the lower social economic status (SES) population as all of our study subjects were commercially insured and represent only 10% of the national youth population. Moreover, by requiring continuous enrollment prior to treatment initiation, our study further excluded lower income patients who may be more likely to have gaps in healthcare coverage. It has been widely reported that the burden of obesity, prediabetes, and T2DM excessively affects children from lower SES families [9,11,14,32,33]. Because lower SES communities are underrepresented in our study, we are unable to extrapolate our results to the general population but can assume our reported rates of treatment initiation are an underestimation of the rates in the overall population. Second, we were unable to analyze additional patient characteristics that may impact OAD prescribing, such as race/ethnicity, family SES status and BMI, as these data are not routinely collected by health insurance plans. Third, outpatient pharmacy claims do not include the identity or specialty of the provider that ordered the filled prescription. Thus, we employed a method that included using a 3-month look-back window in outpatient claims files to classify patients who visited either a family practitioner or pediatrician and separately considered those patients who visited both or other specialties. Although this method does not guarantee the correct classification of the prescribing provider, using a 6-month look back window resulted in consistent findings with the 3-month window. Lastly, prescription claims data do not capture medications obtained without insurance, such as drugs paid out-of-pocket and is of particular concern to our study given that $4 generic drug programs launched in late 2006, offering metformin at discounted prices (not resulting in insurance claims). Although the impact of these low-cost, out-of-pocket programs on claims for OADs is unknown[34], it has been reported that at least 1 in 10 warfarin prescriptions are filled in this manner[35]. This may partially explain the observed decrease in OAD usage from 2008 to 2012.

## Conclusion

The results from our study demonstrated an increase in OAD initiation among children and adolescents between 2002 and 2008. Furthermore, time trends from 2002 to 2012 consistently showed higher rates of prescriptions by FPs compared to general pediatricians. We observed a decrease in OAD initiators beginning 2008, which is likely multi-factorial, reflecting a decreased burden of obesity in the population along with prescriptions filled without health insurance coverage. Continued efforts to educate and support physicians treating these patients are necessary in order to address the emerging epidemic of T2DM and its consequences in youth.
